# PeRsonalIsed MEdicine in Rheumatoid Arthritis (PRIMERA) trial: a multicentre, open-label, randomised controlled trial comparing routine care with a tailor-made approach

**DOI:** 10.1136/bmjopen-2025-108887

**Published:** 2025-12-18

**Authors:** Hamit Harun Dag, Agnes E M Looijen, Harald E Vonkeman, Annemiek Willemze, Lindy-Anne Korswagen, Roos C Padmos, Floris A van Gaalen, Ilja Tchetverikov, Jos H van der Kaap, Josien J Veris-van Dieren, Naghmeh Riyazi, Julia Spierings, Annette H M van der Helm-van Mil, Pascal H P de Jong

**Affiliations:** 1 Rheumatology, Erasmus Medical Center, Rotterdam, The Netherlands; 2 Department of Rheumatology and Clinical Immunology, Medisch Spectrum Twente, Enschede, The Netherlands; 3 Department of Psychology, Health and Technology, University of Twente, Enschede, The Netherlands; 4 Rheumatology and Clinical Immunology, Maasstad Hospital, Rotterdam, The Netherlands; 5 Rheumatology, Franciscus Gasthuis en Vlietland, Rotterdam, The Netherlands; 6 Rheumatology, IJsselland Hospital, Capelle aan den IJssel, The Netherlands; 7 Rheumatology, Leiden University Medical Center, Leiden, The Netherlands; 8 Rheumatology, Albert Schweitzer Hospital, Dordrecht, The Netherlands; 9 Rheumatology, Admiraal De Ruyter Hospital, Goes, The Netherlands; 10 Rheumatology, Reumazorg ZWN, Roosendaal, The Netherlands; 11 Rheumatology, Haga Hospital, The Hague, The Netherlands; 12 Department of Rheumatology and Clinical Immunology, UMC Utrecht, Utrecht, The Netherlands

**Keywords:** rheumatology, randomized controlled trial, disease management, treatment outcome, health care costs, patient reported outcome measures

## Abstract

**Introduction:**

Rheumatoid arthritis (RA) is a heterogeneous disease, which current treatment guidelines insufficiently accommodate, as they predominantly emphasise the suppression of disease activity. However, a step towards personalised medicine is preferred to further optimise treatment and requires homogeneous subgroups with similarities in pathophysiological mechanisms and treatment responses. Prior research has already demonstrated notable differences in the pathophysiology of patients with autoantibody-positive and autoantibody-negative RA, as well as differences in treatment responses, which may serve as a strong basis for personalised medicine. Additionally, there is evidence suggesting that an early treatment response is indicative of future courses. Based on these findings, we designed a personalised medicine trial in RA that compares the effectiveness and cost-effectiveness of a tailor-made approach with routine care.

**Methods and analysis:**

The PeRsonalIsed Medicine in RA (PRIMERA) trial is a multicentre, open-label, randomised controlled trial that includes 300 adult patients with newly diagnosed, DMARD-naïve RA, according to 2010 American College of Rheumatology/EULAR criteria. Patients are randomised into either routine care or a tailor-made approach. Both management approaches use a treat-to-target strategy, aiming for low disease activity (LDA, Disease Activity Score using 44 joints (DAS) ≤2.4). In routine care, initial treatment consists of methotrexate along with a single intramuscular dose of glucocorticoids (GCs) and treatment can be intensified after 3, 7 and 10 months if LDA is not reached. Conversely, initial treatment in the tailor-made approach depends on the presence of autoantibodies, with patients with autoantibody-positive and autoantibody-negative RA starting with hydroxychloroquine or methotrexate together with a single intramuscular dose of GCs, respectively. Medication intensifications will be allowed at months 1, 3, 4, 7 and 10. Intensifications at months 1 and 4 depend on whether patients have an early sufficient response to GCs and targeted synthetic disease-modifying antirheumatic drugs (tsDMARDs), respectively. The tailor-made approach is superior to routine care if no more biological DMARDs (bDMARDs) or tsDMARDs are used after 10 months of treatment, while the mean DAS over time is lower. Our primary outcome is the proportional difference in bDMARD or tsDMARD usage after 10 months of treatment between routine care and the tailor-made approach. Secondary outcomes are DAS over time, time to achieve LDA, cost-effectiveness and patient-reported outcome measurements over time.

**Ethics and dissemination:**

Ethical approval has been granted by Erasmus MC Medical Ethics Review Committee (MEC-2020-0825). The results will be disseminated through peer-review journals and medical congresses.

**Trial registration number:**

ISRCTN16170070.

**STRENGTHS AND LIMITATIONS OF THIS STUDY**

- The randomised controlled clinical trial design provides a higher level of evidence.
- Unlike complex omics-based strategies, the proposed tailor-made approach is simple to implement in daily practice.
- Although an open-label trial introduces a potential risk of bias, the unconventional tailor-made approach makes it unlikely that clinicians will adopt it as standard practice during the study period.
- The 10-month study period limits assessment of long-term outcomes.

## Introduction

 Clinical and radiographic outcomes in rheumatoid arthritis (RA) have improved enormously over the last two decades due to early detection of the disease, early initiation of disease-modifying antirheumatic drugs (DMARDs) and a treat-to-target management approach.^1^,^3^ Although RA is a heterogeneous disease, current treatment guidelines insufficiently reflect this diversity, as they predominantly prioritise the reduction of disease activity rather than tailoring interventions to underlying pathogenic mechanisms.^2^,^4^ To better fit the heterogeneous nature of the disease, a step towards personalised medicine could further improve our rheumatic care, but requires homogeneous subgroups with similarities in pathophysiological mechanisms and treatment responses.^5^,^8^

While the pathophysiological mechanisms defining RA subpopulations still need to be elucidated, existing biomarkers may serve as a framework towards personalised medicine. Specifically, two RA subtypes are distinguished based on the presence or absence of autoantibodies, anticitrullinated protein antibody (ACPA) and/or rheumatoid factor (RF), which could serve as the first pillar of a tailor-made approach.^9 10^ Autoantibody-positive RA has a worse prognosis, more often has a destructive course, is related to functional impairment, requires intensive treatment, including the use of biological DMARDs (bDMARDs), and has a lower chance of achieving sustained DMARD-free remission.^9^,^13^ These disparities in clinical outcomes are supported by underlying pathogenetic differences. Previous research has demonstrated differences in the distribution of T-cell and B-cell populations in blood, lymph nodes and synovial tissue, as well as differences in B-cell receptor signalling and immune profiles.^14^,^17^ Patients with autoantibody-positive RA, for example, have an upregulation of genes associated with adaptive immunity, while genes related to the function of innate immune cells show an upregulation in autoantibody-negative patients.^16^ Aforementioned reasoning could also plausibly clarify the difference in treatment response to specific B-cell or T-cell targeted therapies.^4 18 19^ For example, patients with autoantibody-positive RA have a better response to rituximab, a B-cell depletion therapy, than patients with autoantibody-negative RA.^20^ Thus, although it remains elusive whether the antibodies themselves are pathogenic, they are an indicator of distinct pathological processes. In support of differences in treatment response, we recently demonstrated that patients with autoantibody-negative RA can be treated with hydroxychloroquine (HCQ) with similar efficacy to methotrexate (MTX), in contrast to autoantibody-positive RA.^21^

A second pillar to stratify patients into subgroups is the presence or absence of a rapid treatment response after DMARD initiation. A delayed treatment response has been shown to increase the risk of erosive disease and functional impairment and is associated with a worse quality of life over time.^22^ Moreover, Luurssen-Masurel *et al* demonstrated that achieving low disease activity (LDA)/remission within 6 months was correlated with less bDMARD use and an increased likelihood of attaining DMARD-free remission.^12^ Patients with RA who promptly reach LDA/remission have different molecular profiles compared with those who do not, for instance, several matrix metalloproteinases, homocysteine levels and B-cell subpopulations differ.^23^,^25^ Because molecular profiles are not feasible to assess in daily practice, a clinical proxy is warranted. The early glucocorticoid (GC) response, measured within 1 month, is a suitable proxy. A poor early GC response is strongly associated with a higher likelihood of not responding to the initial conventional synthetic (cs)DMARD therapy.^26^

The third pillar to note is the rapid response to tsDMARDs. Although tumour necrosis factor inhibitors (TNFi) are often the first choice of bDMARD, because of clinical experience and long-term data, the optimal effect of TNFi can take up to 6 months and about one-third of patients will have an inadequate response.^27^ The finding that early disease is more responsive to treatment than in later stages underscores the need to find the right bDMARD or tsDMARD as quickly as possible.^28 29^ Since tsDMARDs have a faster treatment effect, an early inadequate tsDMARD response can be used as an indication to switch to a bDMARD.^30^ When initial csDMARDs fail and other DMARDs are needed, this step allows for an additional layer towards personalised treatment in the management of RA.

The aforementioned pillars towards personalised medicine in RA—(1) presence of autoantibodies, (2) early GC response and (3) fast tsDMARDs effect—have never been combined into one single approach, nor has their integrated effectiveness been compared with routine care. We hypothesise that the proposed tailor-made approach, in which treatment decisions are based on these pillars, will result in no more bDMARD or tsDMARD usage while the mean disease activity over time is lower. Therefore, our primary objective is to compare the proportional difference in bDMARD or tsDMARD use after 10 months of treatment between the tailor-made approach and routine care in patients with newly diagnosed, DMARD-naïve RA. Secondary outcomes are disease activity over time, time to achieve LDA, cost-effectiveness and patient-reported outcome measurements (PROMs) over time, including functional ability, quality of life, fatigue and pain.

## Methods and analysis

### Trial design and setting

The PeRsonalIsed MEdicine in RA (PRIMERA) trial is a multicentre, open-label, randomised controlled trial that compares the effectiveness and cost-effectiveness of a tailor-made approach with routine care. The trial, including patient recruitment, follow-up and data cleaning, is carried out in three academic hospitals, seven general hospitals and one specialised rheumatology centre between December 2019 and December 2025.

### Recruitment and eligibility criteria

#### Recruitment

Patients with newly diagnosed RA—defined as individuals who meet the 2010 American College of Rheumatology (ACR)/EULAR classification criteria for RA for the first time, and have not previously received DMARD therapy—will be screened for eligibility, irrespective of the symptom duration. Patients may withdraw from the study at any time without any consequences. The investigator may also withdraw patients deemed ineligible. No replacement will be made for subjects withdrawing from the trial after randomisation.

#### Inclusion criteria

- Patients with newly diagnosed, DMARD-naïve RA according to 2010 ACR/EULAR criteria for RA.^31^
- Age ≥18 years.

#### Exclusion criteria

- Current or previous DMARD usage.
- Systemic GC use within the 3 months prior to randomisation.
- Unable to understand, speak and write in Dutch.
- (Relative) contraindications for study medication, among others:
  - Recent infection or malignancy within the 3 months prior to inclusion.
  - Pregnancy or lactation.
  - Female patients of childbearing potential and male patients whose partner is of childbearing potential who are not willing to ensure that they or their partner use effective contraception during the trial and for 3 months thereafter.
  - History of clinically significant hepatic dysfunction, as indicated by abnormal liver function tests. At inclusion, any single parameter may not exceed two times the upper limit of normal.
  - History of renal injury, glomerulonephritis, subjects with one kidney or a glomerular filtration rate <30 mL/min.

### Randomisation and blinding

Patients are randomised with a 1:1 ratio to either the tailor-made approach or routine care using minimisation randomisation stratified by centre, using ALEA clinical software.^32^ Blinding will not be applied to physicians, patients or research nurses, because all DMARDs are used according to label and are prescribed by the treating rheumatologists.

### Interventions

#### Management approaches

In routine care, patients will initiate treatment with MTX and a single intramuscular dose of GCs. In the tailor-made approach, initial treatment will be stratified by autoantibody status: autoantibody-negative patients (ACPA-negative and RF-negative) will receive HCQ and a single dose of intramuscular GCs, while autoantibody-positive patients (ACPA-positive and/or RF-positive) will start with MTX and a single dose of intramuscular GCs.

**Figure 1:**
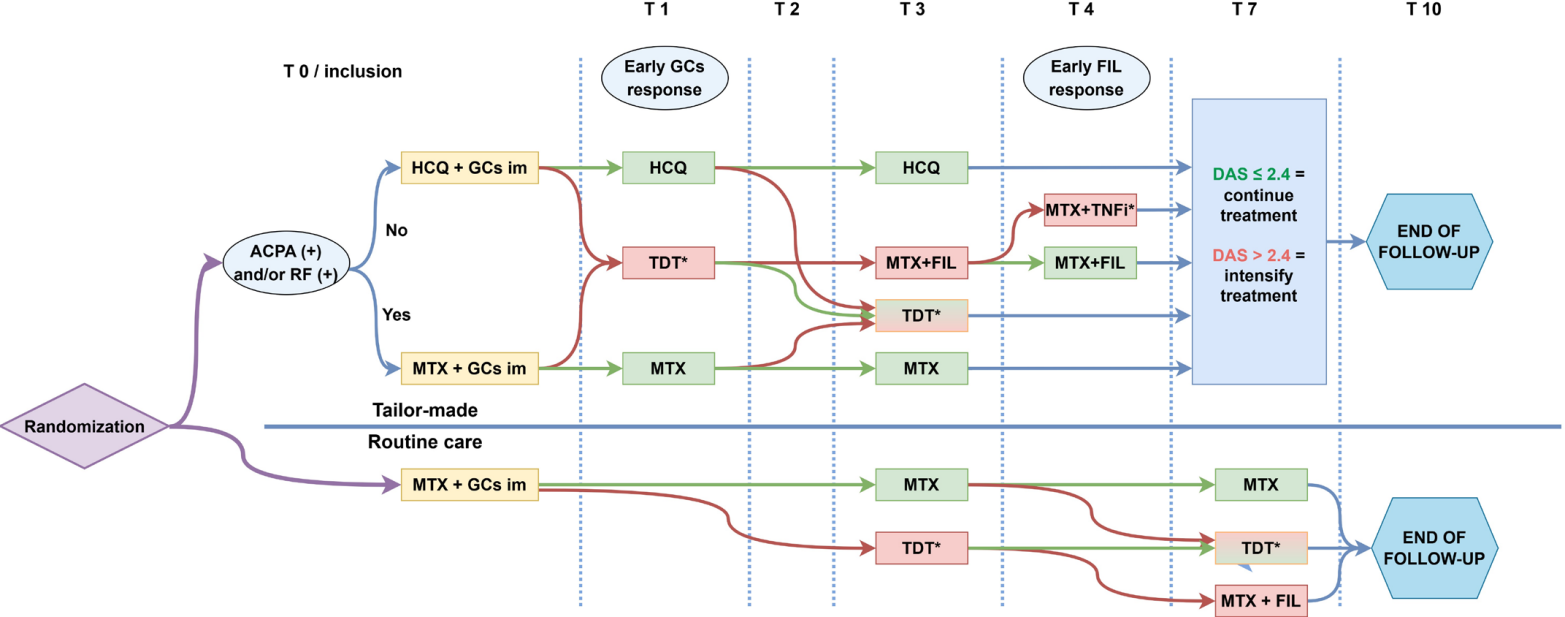
Medication protocol. *An optional single dose of intramuscular GCs can be given when treatment is intensified to TDT or MTX+TNFi. MTX may be replaced with leflunomide when discontinuation is necessary. ACPA, anticitrullinated protein antibody; DAS, Disease Activity Score; FIL, filgotinib; GC, glucocorticoid; HCQ, hydroxychloroquine; im, intramuscular; MTX, methotrexate; RF, rheumatoid factor; TDT, triple DMARD therapy (consisting of MTX, sulfasalazine and hydroxychloroquine); TNFi, tumour necrosis factor inhibitor.

Both management approaches follow a treat-to-target strategy, aiming for LDA (Disease Activity Score using 44 joints (DAS) ≤2.4).^33^ If DAS >2.4, treatment is intensified per protocol until LDA is reached. The intensification steps for both treatment approaches occur in the following order: (1) triple DMARD therapy, consisting of MTX, sulfasalazine and HCQ combined with an optional single dose of intramuscular GCs; (2) MTX and filgotinib (FIL); (3) MTX and TNFi combined with an optional single dose of intramuscular GCs and (4) MTX and second TNFi combined with an optional single dose of intramuscular GCs. In routine care, medication can be intensified after 3, 7 and 10 months. In the tailor-made approach, additional intensification steps can occur after 1 and 4 months, depending on the response to intramuscular GCs and FIL, respectively. A good response to intramuscular GCs and FIL is defined as a DAS ≤2.4. Figure 1 outlines the treatment protocol.

DMARD and GC dosages are given in table 1. All prescribed medications in this trial are approved for use in RA and are used according to their label. Safety monitoring will be carried out according to Dutch guidelines and includes scheduled laboratory monitoring.^34^,^37^ In the case of (serious) adverse events ((S)AEs), using WHO adverse reaction terminology, treatment may be discontinued, adjusted for dose or switched per protocol at the discretion of the treating rheumatologist.^38^ MTX may be administered subcutaneously in case of gastrointestinal intolerance or may be replaced with leflunomide when discontinuation is necessary.

**Table 1:** Medication dosages and intervention overview

Drug	Dosage	Administration
Methotrexate	Week 1: 15 mg one time a week Week 2: 20 mg one time a week Week ≥3: 25 mg one time a week	Oral (or subcutaneous)
Hydroxychloroquine	400 mg once per day and after 3 months 200 mg once per day	Oral
Sulfasalazine	Week 1: 500 mg two times per day Week 2: 500 mg three times a day Week ≥3: 1000 mg two times per day	Oral
Leflunomide	20 mg once per day	Oral
Triamcinolone acetonide	80 mg (once)	Intramuscular
Methylprednisolone	120 mg (once)	Intramuscular
TNF inhibitors Adalimumab Certolizumab pegol Etanercept Golimumab Infliximab	40 mg one time per 2 weeks 400 mg at 0, 2 and 4 weeks, thereafter 200 mg one time per 2 weeks 50 mg one time a week 50 mg one time per month 3–5 mg/kg at 0, 2 and 6 weeks, thereafter one time per 8 weeks	Subcutaneous Subcutaneous Subcutaneous Subcutaneous Intravenous
Filgotinib	200 mg once per day	Oral

#### Early escalation

Early escalation is defined as advancing to the next step in the medication protocol prior to the scheduled study visit intended to assess the treatment target (DAS ≤2.4). Early escalation is allowed in case of very active disease requiring immediate intensification, as determined by the treating rheumatologist. In the case of early escalation, an unscheduled study visit will be arranged by the treating rheumatologist and/or research nurse and the patient will be invited to come to the outpatient clinic. Follow-up visits will be scheduled in accordance with the timeline specified in the study protocol. No medication escalation will be advised at the subsequent scheduled visit following the early escalation.

#### Use of co-intervention (if applicable)

No DMARDs other than those specified in the protocol are permitted during the study follow-up. Oral GCs are prohibited; intramuscular GCs are allowed only at inclusion and as bridging therapy when intensifying to triple DMARD therapy or TNFi. Intra-articular GC injections are permitted during follow-up with a maximum of two per 3 months. Standard-dose non-steroidal anti-inflammatory drugs (NSAIDs) are allowed as co-treatment or rescue medication. There are no explicitly excluded concomitant medications. However, drugs contraindicated with study treatments must be avoided. New therapies for comorbid conditions should only be initiated if deemed safe in combination with the study medication.

#### Escape medication

Escape medication is defined as any new therapeutic intervention, or a significant modification of ongoing therapy initiated due to lack of efficacy or clinical worsening. Only the following prespecified escape medications are permitted: (1) intra-articular GCs with a maximum of two injections per 3 months and (2) NSAIDs. The use of intra-articular GCS will be registered. The use of NSAIDs will not be documented.

#### Protocol violation

A protocol violation occurs when the rheumatologist deviates from the predefined medication protocol. If a rheumatologist chooses not to escalate or continue treatment according to the DAS, a follow-up visit is scheduled after 2 weeks to verify the DAS. Persistent non-compliance constitutes a protocol violation. Additionally, administration of rescue medication outside the prespecified options also qualifies as a protocol violation. If a protocol violation occurs, the patient may remain in the study, and subsequent procedures will continue in accordance with the study protocol. If treatment intensification is not carried out at the scheduled time point, it will be implemented at the next step, provided there is ongoing evidence of active disease.

### Adverse events

SAEs are defined as any undesirable experience occurring to a patient during the study, whether or not considered related to the study medication. An SAE is any unexpected medical occurrence or effect that:

- results in death;
- is life threatening (at the time of the event);
- requires hospitalisation or prolongation of existing inpatients’ hospitalisation;
- results in persistent or significant disability or incapacity;
- is a congenital anomaly or birth defect;
- any other important medical event that did not result in any of the outcomes listed above due to medical or surgical intervention, but could have been based on appropriate judgement by the investigator.

An elective hospital admission unrelated to the study procedures will not be classified as an SAE. All (S)AEs reported spontaneously by the patient or observed by the investigator or study staff will be documented. Each event will be followed until resolution or stabilisation. Follow-up may include additional diagnostic tests, medical procedures or referral to a general practitioner or specialist if needed. (S)AEs will be documented at each study visit through patient questionnaires and physician reports. Patient-reported events include infections (commonly upper respiratory), headache, nausea, diarrhoea, vomiting and injection site reactions. Physician-reported events include hypertension, infections, neutropenia/leucopenia and liver function test abnormalities.

### Participant timeline

Patients will be assessed every month during the first 4 months and at months 7 and 10. DAS will be calculated by a trained research nurse. At each visit and 2 weeks after inclusion, patients will also complete online questionnaires. Additional blood samples will be taken at baseline, 1 and 3 months and at 2 and 4 months if DAS >2.4 at the previous visit. See also table 2 and figure 1 for an extensive timeline overview.

**Table 2:** Schedule of the trial and data collection overview

		Study period							
	Enrolment	Allocation	Postallocation						Close-out
Time point	−*t*_*1*_	0	*t*_*1/2*_	*t*_*1*_	*t*_*2*_	*t*_*3*_	*t*_*4*_	*t*_*7*_	*t*_*10*_
Enrolment									
PIF		X							
Eligibility screen	X								
Informed consent		X							
Allocation		X							
Assessments (hospital)									
Demographic		X							
History		X							
Length, weight and BMI		X							
Comorbidity		X							
Physical examination by RN		X		X	X	X	X	X	X
Global assessment by patient and RN		X		X	X	X	X	X	X
Adverse events (self-reported)				X	X	X	X	X	X
Hospital admission in past 3 months						X		X	X
Routine laboratory tests		X		X	X	X	[X]	X	X
Additional blood samples (serum (2×4.5 mL) and heparin (6×8 mL) tube and Paxgene blood RNA tubes)		X		X	(X)	X	(X)	(X)	
Assessments (online questionnaires)									
General (smoking, alcohol and drugs)		X							
Disease activity compared with previous visit				X	X	X	X	X	X
RAPID3		X	X	X	X	X	X	X	X
Morning stiffness (severity and duration)		X		X	X	X	X	X	X
GH (VAS)		X		X	X	X	X	X	X
Fatigue (VAS)		X		X	X	X	X	X	X
Pain (NRS)		X	X	X	X	X	X	X	X
Pain (GPQ)		X		X	X	X	X	X	X
Pain (PainDETECT)		X		X	X	X	X	X	X
HAQ (functional ability)		X		X	X	X	X	X	X
EQ-5D (quality of life)		X		X	X	X	X	X	X
WPAI (work and productivity)		X				X		X	X
Medical consumption (MCQ)						X		X	X
Patient satisfaction via VAS and TSQM				X		X		X	X
MARS-5 (medication adherence) *(30 s)*				X		X		X	X
Patient-SDM				X		X		X	X
Participation (IPAQ)		X							X

(X) Collection will only take place in case of DAS >2.4 at previous visit.

[X] Depending on medication changes at visit T3.

Adopted from SPIRIT, the schedule of enrolment, interventions and assessments template.^55^

BMI, body mass index; EQ-5D, EuroQol with 5 dimensions and 5 levels; GH, general health; GP, general practitioner; GPQ, Generalised Pain Questionnaire; HAQ, Health Assessment Questionnaire; IPAQ, Impact on Participation and Autonomy Questionnaire; MARS-5, Medication Adherence Report Scale; MCQ, Medical Consumption Questionnaire; NRS, Numeric Rating Scale; PIF, patient information folder; RAPID3, Routine Assessment of Patient Index Data 3; RN, research nurse; SDM, Shared Decision-Making Questionnaire; SPIRIT, Standard Protocol Items: Recommendations for Interventional Trials; TSQM, Treatment Satisfaction Questionnaire for Medication; VAS, visual analogue scale; WPAI, Work Productivity and Activity Impairment.

### Primary end point

The primary end point is the proportional difference in bDMARD or tsDMARD usage after 10 months of treatment between the tailor-made approach and routine care. The tailor-made approach is superior to routine care if no more bDMARDs or tsDMARDs are used, while the mean DAS over time is lower.

### Secondary end points

Our secondary end points are DAS over time, time to achieve LDA, cost-effectiveness and PROMs over time, including functional ability, quality of life, fatigue and pain.

The DAS is a pooled index that involves the incorporation of a graded 53-joint count for tenderness (Ritchie Articular Index (RAI)), a 44-joint count for swelling (SJC44), erythrocyte sedimentation rate (ESR) and general health (GH, measured with a visual analogue scale (VAS) 0–100 mm) into a formula to obtain a numerical indicator of disease activity.^33^ The DAS formula is 0.53938√(RAI)+0.06465(SJC44)+0.33ln(ESR)+0.00722(GH). Thresholds for remission and LDA are <1.6 and ≤2.4, respectively.

The cost-effectiveness analysis uses the incremental cost-effectiveness ratio (ICER), defined as the ratio of the difference in costs to the difference in incremental benefits between the two management strategies, as outcome. Cost-effectiveness will be evaluated from both societal (direct and indirect costs) and healthcare perspectives (only direct costs).^39^ Direct costs will be assessed every 3 months using the iMTA Medical Consumption Questionnaire.^40^ Indirect costs will be measured using the Work Productivity and Activity Impairment (WPAI) questionnaire.^41^ The incremental benefits are determined by quality-adjusted life years.^42 43^

Functional ability is measured with the Health Assessment Questionnaire (HAQ) and higher scores indicate a poorer function.^44 45^ Quality of life, measured with Dutch 5-level EQ-5D, with a score of 1 representing the best health state and lower values representing worse health states.^42 43^ Fatigue is measured with a VAS (0–100 mm) and pain is measured with a Numeric Rating Scale (NRS, 0–10). In both, the question asks the severity of the last week, and higher scores reflect greater severity.

### Exploratory end points

Other outcomes will include end points that reflect both clinical and patient viewpoints at specific time points and over time.

#### Clinical outcomes

- Disease activity (states) at 10 months, measured with the DAS.

#### Patient-reported outcome measurements

- Functional ability, measured with the HAQ-Disability Index.^44 45^
- Quality of life, measured with the Dutch 5-level EQ-5D.^42 43^
- Fatigue, measured with a VAS (0–100 mm).
- Pain, measured with an NRS (0–10), Generalised Pain Questionnaire and PainDETECT questionnaire.^46 47^
- Self-reported disease activity, measured with the Routine Assessment of Patient Index Data 3.^48^
- Morning stiffness severity and duration, measured with an NRS (0–10).
- GH, measured with a VAS (0–100 mm).
- Patient satisfaction with medical treatment, measured with the Treatment Satisfaction Questionnaire for Medication and a VAS (0–100 mm).^49^
- Patient compliance, measured with the Medication Adherence Report Scale.^50^
- Patient participation, measured with the Shared Decision-Making Questionnaire.^51^
- Patient autonomy and participation, measured with the Impact on Participation and Autonomy questionnaire.^52^
- Worker productivity, measured with the WPAI.^41^

#### Other study parameters

At baseline, we will gather data on demographics and comorbidities. The demographic dataset will include age, sex, ethnicity, marital status, work status, smoking status and alcohol consumption as well as information on disease duration. Furthermore, we will ask for the presence of the following comorbidities: diabetes mellitus, inflammatory bowel disease, cardiovascular diseases, urinary tract disorders and malignancies. During the general physical examination, we will measure the height and weight, with which we will calculate the body mass index.

### Sample size

The tailor-made approach is superior to routine care if no more bDMARDs/tsDMARDs are used, while the mean DAS over time is lower. The PRIMERA trial is powered to detect a 5% non-inferiority difference in proportion of patients using a bDMARD or tsDMARD after 10 months of treatment. Based on theTreatment in the Rotterdam Early Arthritis Cohort (tREACH) trial, the following assumptions were made: (1) in the tailor-made approach, bDMARD or tsDMARD usage will be similar to the initial triple DMARD therapy arms (27%) and (2) in routine care, bDMARD or tsDMARD usage was set at the proportion of patients, randomised to MTX monotherapy who had an active disease at ≥2 out of four visits during the first year of follow-up (36%).^53^ If using a significance level of α=0.05 and a power of 80%, 150 patients are needed for each management approach allowing for 10% dropout.

Besides no more bDMARDs or tsDMARDs after 10 months of treatment, the mean DAS over time should also be lower. Therefore, a second power calculation was performed to verify if enough patients are included. If we again use data from the tREACH trial, where the mean DAS difference over time between initial triple DMARD therapy and MTX monotherapy is −0.21 and if we want to use a multivariable mixed model (MM) with three covariates, a target sample size of 45 patients per management approach will be needed to detect the aforementioned difference with a power of 80% and two-sided α=0.05.

Therefore, we need 300 patients with newly diagnosed, DMARD-naïve RA to confirm that the tailor-made approach is superior to routine care if no more bDMARDs or tsDMARDs are used, while the mean DAS over time is lower.

### Statistical methods

The primary analyses will be conducted according to the intention-to-treat (ITT) principle. In ITT analyses, all patients will be analysed in the groups to which they were originally assigned, regardless of whether they received or adhered to the allocated management approach.

Additionally, end points will be analysed using a per-protocol (PP) analysis. In PP analysis, patients are included if they have adhered sufficiently to the protocol to maintain the heterogeneity of the treatment management approaches between the two groups. Patients will be excluded from this analysis if they:

- used oral or intramuscular GCs outside the treatment protocol;
- underwent unindicated treatment intensifications or early escalations;
- did not intensify treatment according to the protocol;
- used <2 csDMARDs when triple DMARD therapy was indicated;
- used MTX instead of HCQ in case of ACPA and RF negativity in the tailor-made treatment arm.

Patients who, in case of contraindications, used a TNFi instead of FIL will be included in the PP analyses.

Baseline characteristics will be compared between both management approaches to identify any imbalances using the χ² test, Student’s t-test or Wilcoxon rank-sum test. Mixed-effects models will account for stratification by hospital. If baseline imbalances are found in age, sex, symptom duration, autoantibody positivity or DAS, then the analyses will be adjusted accordingly. As we assume that missing data are (completely) at random, these missing data will be handled by our MMs. Therefore, no imputation plan has been made. No subgroup or interim analyses have been planned. Analyses will be performed using STATA V.18 or later, or R V.4.4.3 or later. A p value of ≤0.05 will be considered statistically significant. For multiple testing, a Bonferroni correction will be considered, where the p value is multiplied by the number of tests performed.

#### Primary end point analyses

For the primary end point, we will compare the proportion of patients using a bDMARD or tsDMARD after 10 months of treatment between the tailor-made approach and routine care, using a two proportion Z-test.

#### Secondary end point analyses

A mixed-effects model will be used to compare the DAS over time between both management approaches. Additionally, a Cox proportional hazards model will be used to compare the time to achieve LDA. Both analyses will account for stratification by hospital and will be adjusted for any potential baseline imbalances. Mixed-effects models will be used to compare PROMs over time between the tailor-made approach and routine care.

The ICER will be the end point of the cost-effectiveness analyses. Both total costs and effectiveness will be assessed using MMs. A probabilistic sensitivity analysis will be performed for the estimation of the ICER by bootstrapping with sampling 10 000 times. Results will be plotted in a cost-effectiveness plane, which will be used to estimate the 95% CI of the ICER. Additionally, a cost-effectiveness acceptability curve will be extracted to show the probability of each management approach being cost-effective at different levels of willingness-to-pay thresholds in comparison with each other. The main drivers of cost-effectiveness will be determined through sensitivity analysis.

#### Exploratory end point analyses

Our exploratory outcomes, which included both clinical outcomes and PROMs, will be compared at different time points using a Student’s t-test, χ² test or Wilcoxon rank-sum test, as appropriate. Mixed-effects models will be used to compare these outcomes over time.

### Data collection and management

#### Data collection

Data will be collected electronically using an electronic case report form (eCRF). All patients and a selection of designated and trained research nurses will complete online questionnaires. Patients will receive an email including a link to complete the questionnaires. Patients may complete online questionnaires within a window spanning 1 week before to 1 week after the scheduled appointment. If the questionnaires are not completed within the final 2 days of the designated window, a reminder will be sent by email, and the completion period will be extended by 1 week.

#### Data storage

The software packages and all data are stored on a server hosted by the Sponsor, Erasmus MC. Data are stored for 15 years after the last visit of the last subject who completes the entire follow-up of the trial.

#### Data confidentiality

All data will be handled confidentially, and all medical centres will be asked to sign a confidentiality agreement. In addition, all staff will be trained before having access to the eCRF.

### Data monitoring

A Data Safety Monitoring Board (DSMB) will be appointed to safeguard the interests of the trial patients, to assess the safety and efficacy of the interventions during the trial and to monitor the overall conduct of the trial, protecting its validity and credibility. The DSMB will comprise (medical) experts with experience in strategic trials and/or the investigational treatment.

The DSMB will convene at least annually, from the first patient enrolment until the final follow-up visit. While no formal interim efficacy analyses are planned, the DSMB will review accumulated data stratified by treatment strategy, assess SAEs and consider relevant external evidence. The DSMB may recommend trial modification or termination at any time based on ethical concerns, safety issues or clear evidence of the effectiveness of one of the treatments.

### Patient and public involvement

Patient and public were not involved in the design, conduct, reporting or dissemination of this study.

## Ethics and dissemination

This trial will be conducted in accordance with the principles of the Declaration of Helsinki.^54^ Ethical approval has been granted by the Erasmus MC Medical Ethics Review Committee (METC in Dutch; MEC-2020-0825). All substantial amendments will be reported to the METC and the competent authority. Non-substantial amendments will be documented and retained by the sponsor. This study is prospectively registered with ISRCTN (ISRCTN16170070). The results will be disseminated through peer-reviewed journals and scientific congresses.
