# Supplementary material for: PeRsonalIsed MEdicine in Rheumatoid Arthritis (PRIMERA) trial: a multicentre, open-label, randomised controlled trial comparing routine care with a tailor-made approach
Source: BMJ Open. 2025 Dec 18;15(12):e108887. doi: 10.1136/bmjopen-2025-108887 (PMC12718583; doi:10.1136/bmjopen-2025-108887)
Supplement: Supplementary file 1 [file bmjopen-2025-108887.reviewer_comments.pdf]

PEER REVIEW HISTORY

BMJ Open publishes all reviews undertaken for accepted manuscripts. Reviewers are asked to complete a checklist review form and are provided with free text boxes to elaborate on their assessment. These free text comments are reproduced below.

ARTICLE DETAILS

Title (Provisional)

PeRsonalIzed MEdicine in Rheumatoid Arthritis (PRIMERA) trial: a multicenter, open-label, randomized controlled trial comparing routine care with a tailor-made approach

Authors

Dag, Hamit Harun; Looijen, Agnes Elisabeth Maria; Vonkeman, Harald E.; Willemze, Annemiek; Korswagen, Lindy-Anne; Padmos, Roos C; Van Gaalen, Floris A; Tchetverikov , I.; Van Der Kaap, Jos H; Veris -Van Dieren, Josien J; Riyazi, Naghmeh; Spierings, Julia; van der Helm - van Mil, Annette; de Jong, Pascal Hendrik Pieter

---

VERSION 1 - REVIEW

**Reviewer**	**1**
**Name**	**Provan, Sella**
**Affiliation**	**Diakonhjemmet Sykehus AS, Rheumatology**
**Date**	**25-Sep-2025**
**COI**	**None**

Thank you for the opportunity to review this protocol.

I have the following minor comments:

Abstract: Please revise the Methods section from line 11-12. You "suddenly" mention glucocorticoids which was confusing. Please give a more precise explanation of the study arms.

Introduction:

I do not fully agree that there is “one size fits all” doctrine as mentioned in the introduction. The EULAR guidelines do open up for variations in corticosteroid administration etc. There are also several treatment strategy studies in RA that are not mentioned. The EULAR guidelines also refrain from recommending HCQ due to lack of evidence, while NORD-STAR includes HCQ in one of the csDMARD strategies applied to certain countries. One could expect this to be discussed in the introduction

Methods:

Regarding power analyses and inclusion of patients. How will you endeavour to include a sufficient number of seronegative patients?

Inclusion: What is the definition of newly diagnosed?

How can unscheduled visits be arranged?

Table 2 should include specification of which measures of disease activity will be recorded. As far as I can se, you mention DAS, but what about other measures such as CDAI, SDAI and different measures of remission?

VERSION 1 - AUTHOR RESPONSE

Reviewer 1, dr. Sella Provan, comments addressed:

Abstract:
Please revise the Methods section from line 11-12. You "suddenly" mention glucocorticoids which was confusing. Please give a more precise explanation of the study arms.
Thank you for your advice. A more detailed explanation has been provided in the methods section of the abstract.

Introduction:
I do not fully agree that there is “one size fits all” doctrine as mentioned in the introduction. The EULAR guidelines do open up for variations in corticosteroid administration etc. There are also several treatment strategy studies in RA that are not mentioned. The EULAR guidelines also refrain from recommending HCQ due to lack of evidence, while NORD-STAR includes HCQ in one of the csDMARD strategies applied to certain countries. One could expect this to be discussed in the introduction
Thank you for your valuable feedback. From our perspective, current EULAR guidelines do not propose homogeneous RA subpopulations that receive varied treatment management recommendations. However, we do agree that there is room for some variations e.g., in corticosteroid use. To better reflect our perspective, we modified the sentence in the introduction as follows at p.4 :
“ Although RA is a heterogeneous disease, current treatment guidelines insufficiently reflect this diversity, as they predominantly prioritize the reduction of disease activity rather than tailoring interventions to underlying pathogenic mechanisms [2-4].”

We recognize that NORD-STAR trial has a significant impact on improving rheumatic care, including the use of HCQ in csDMARD combination therapies. However, in our trial, we hypothesize that autoantibody negative patients can initially be treated with HCQ monotherapy with similar efficacy to MTX monotherapy. Since the NORD-STAR trial does not include a group that uses HCQ monotherapy, we cannot highlight the NORD-STAR trial

as evidence supporting our hypothesis. However, we referenced this trial in the introduction section as you advised as follows at p.4:
" To better fit the heterogeneous nature of the disease, a step towards personalized medicine could further improve our rheumatic care, but requires homogeneous subgroups with similarities in pathophysiological mechanisms and treatment responses [5-8]."

Methods:
Regarding power analyses and inclusion of patients. How will you endeavor to include a sufficient number of seronegative patients?
Thank you for your question. We do not have any subgroup analysis planned on autoantibody negative patients (Statistical methods section, p.15). Therefore, no additional power analyses on a subgroup of patients have been conducted.

Inclusion: What is the definition of newly diagnosed?
Thank you for raising this point. We modified Recruitment section on p.6 as follows:
" Newly diagnosed RA patients —defined as individuals who meet the 2010 ACR/EULAR classification criteria for RA for the first time, and have not previously received DMARD-therapy—will be screened for eligibility, irrespective of the symptom duration."

How can unscheduled visits be arranged?
Thank you for your question. We have provided more details on how unscheduled visits will be arranged. Please see the Early escalation section on p.9.
" Early escalation is allowed in case of very active disease requiring immediate intensification, as determined by the treating rheumatologist. In case of early escalation, an unscheduled study visit will be arranged by the treating rheumatologist and/or research nurse and the patient will be invited to come to the outpatient clinic. Follow-up visits will be scheduled in accordance with the timeline specified in the study protocol."

Table 2 should include specification of which measures of disease activity will be recorded. As far as I can see, you mention DAS, but what about other measures such as CDAI, SDAI and different measures of remission?
Within our dataset we have the opportunity to calculate/report the CDAI and SDAI, but they are not part of our analysis plan. In our analysis plan only the DAS will be included as a secondary outcome. For this reason other disease activity measures such as CDAI and SDAI have not been presented in Table 2.

VERSION 2 - REVIEW

**Reviewer**	**1**
**Name**	**Provan, Sella**
**Affiliation**	**Diakonhjemmet Sykehus AS, Rheumatology**
**Date**	**17-Nov-2025**

COI

Thank you for the revision. I have no further comments
